# *Daphnia* as a Sentinel Species for Environmental Health Protection: A Perspective on Biomonitoring and Bioremediation of Chemical Pollution

**DOI:** 10.1021/acs.est.2c01799

**Published:** 2022-09-28

**Authors:** Muhammad Abdullahi, Xiaojing Li, Mohamed Abou-Elwafa Abdallah, William Stubbings, Norman Yan, Marianne Barnard, Liang-Hong Guo, John K. Colbourne, Luisa Orsini

**Affiliations:** † Environmental Genomics Group, School of Biosciences, the University of Birmingham, Birmingham B15 2TT, U.K.; ‡ School of Geography, Earth and Environmental Sciences, the University of Birmingham, Birmingham B15 2TT, U.K.; § Department of Biology, York University, and Friends of the Muskoka Watershed, Bracebridge, Ontario P1L 1T7, Canada; ∥ Institute of Environmental and Health Sciences, China Jiliang University, 258 Xueyuan Street, Hangzhou, Zhejiang 310018, People’s Republic of China; ⊥ The Alan Turing Institute, British Library, 96 Euston Road, London NW1 2DB, U.K.

**Keywords:** chemical mixtures, bioremediation, monitoring, water flea, water pollution, omics

## Abstract

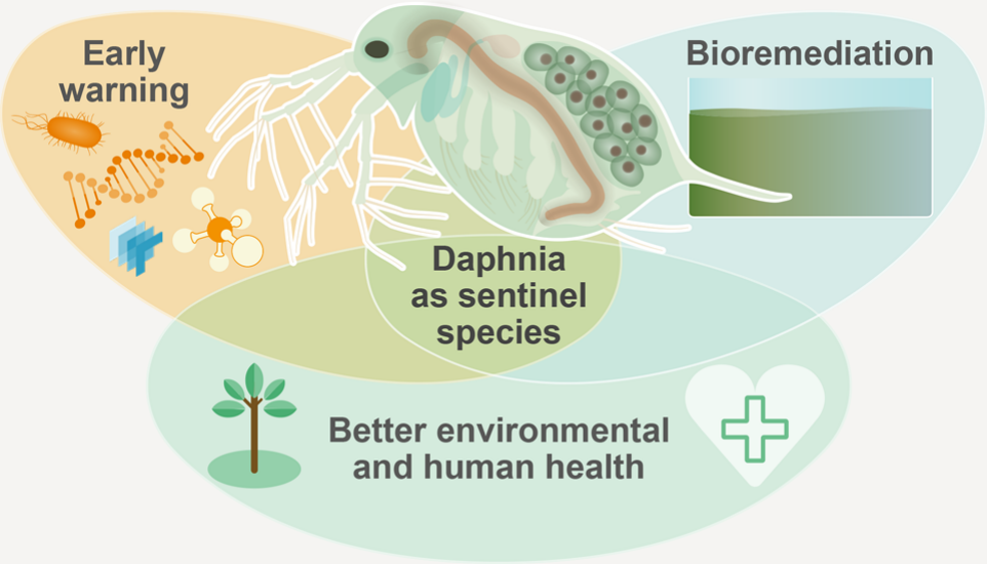

Despite available technology and the knowledge that chemical pollution damages human and ecosystem health, chemical pollution remains rampant, ineffectively monitored, rarely prevented, and only occasionally mitigated. We present a framework that helps address current major challenges in the monitoring and assessment of chemical pollution by broadening the use of the sentinel species *Daphnia* as a diagnostic agent of water pollution. And where prevention has failed, we propose the application of *Daphnia* as a bioremediation agent to help reduce hazards from chemical mixtures in the environment. By applying “omics” technologies to *Daphnia* exposed to real-world ambient chemical mixtures, we show improvements at detecting bioactive components of chemical mixtures, determining the potential effects of untested chemicals within mixtures, and identifying targets of toxicity. We also show that using *Daphnia* strains that naturally adapted to chemical pollution as removal agents of ambient chemical mixtures can sustainably improve environmental health protection. Expanding the use of *Daphnia* beyond its current applications in regulatory toxicology has the potential to improve both the assessment and the remediation of environmental pollution.

## Abstract 2

Chemical pollution damages human and ecosystem health. We present a framework that broadens the use of the sentinel species *Daphnia* as a diagnostic and bioremediation agent of water pollution.

## Introduction

The use of animals as sentinels to detect threats to human health dates to the era when coal miners brought caged canaries into mines to provide early warning of toxic gases. The concept of the “canary in the coal mine” is based on three principles: the sentinel species (i) is more sensitive than both humans and most other animals to toxic exposure, (ii) shares the same environment as humans, and (iii) produces a readily detectable effect of the toxic exposure.^1^ Following the sentinel species model, hazard is typically characterized in surrogate models and extrapolated to the target species; human hazard is traditionally characterized using surrogate mammalian species, whereas ecological hazard is characterized by exposing representative species of key taxonomic groups (e.g., primary producers, invertebrates, and vertebrate embryos) to the reputed hazards.^2^

Despite the acknowledged value of sentinel species to detect hazards, their full potential as indicators of threats to humans and the environment has not been fully realized. With the One-Medicine-One-Health initiative which began in 2010, stemming from the One-Health concept,^3^ the nexus between humans, other animals, and the environment was endorsed by physicians and veterinarians but did not lead to substantial changes in the way environmental health hazard is assessed.^4,5^

Anthropogenic chemicals used in most production processes are transported globally and usually end up in the environment as unintentional pollutants that may harm humans and damage the environment.^6−8^ Until the last few decades, industrial chemicals were not routinely assessed for their risk and impact on wildlife and humans^9^ and measurements of toxicity were not always part of premarket screening for chemical safety.^10^ Even the most up-to-date national inventories do not include chemical mixtures or byproducts and degradation products of the parent compounds that are released into the environment.^11^ As a result, more than 235 000 individual chemicals and 120 000 unregulated mixtures have been found in the environment.^8,12^ Chemicals entering the environment can bioaccumulate in animal tissue and be biomagnified through the trophic chain, eventually entering our food supply, and causing adverse health outcomes, even at low doses (e.g., refs (7), (12), and (13)). Chemical cocktails of unknown mixtures can interact with other environmental factors (e.g., climate change, microplastics, and increased salinity) collectively contributing to environmental degradation^14^ and causing the premature death of 9 million people every year (16% of deaths worldwide).^7,15^ Chemical pollution, together with overexploitation of resources, land use, and climate change, is one of the main causes of loss of biodiversity and has led to the deterioration of 60% of ecosystem services worldwide in the last few decades.^14,16,17^

The current one-chemical-at-a-time, hazard-focused, and siloed approach to environmental and human health protection is insufficient to address these interconnected and interdependent challenges. On one hand there is a need for a better diagnosis of the impact of chemicals on wildlife and humans. On the other, when chemicals have entered the environment, remediation may be the only solution to reduce preventable health effects and deaths. Sentinel species can play the dual role of diagnostic and remedial agents of chemical pollution. In this Perspective, we present a framework that expands the use of the sentinel species *Daphnia* to act both as a diagnostic early warning system and as a bioremediation tool for environmental pollution. We identify the outstanding challenges in modern (eco)toxicology that can be mitigated with the application of the framework.

## Broadening the Use of the Sentinel Species *Daphnia*

**Figure 1:**
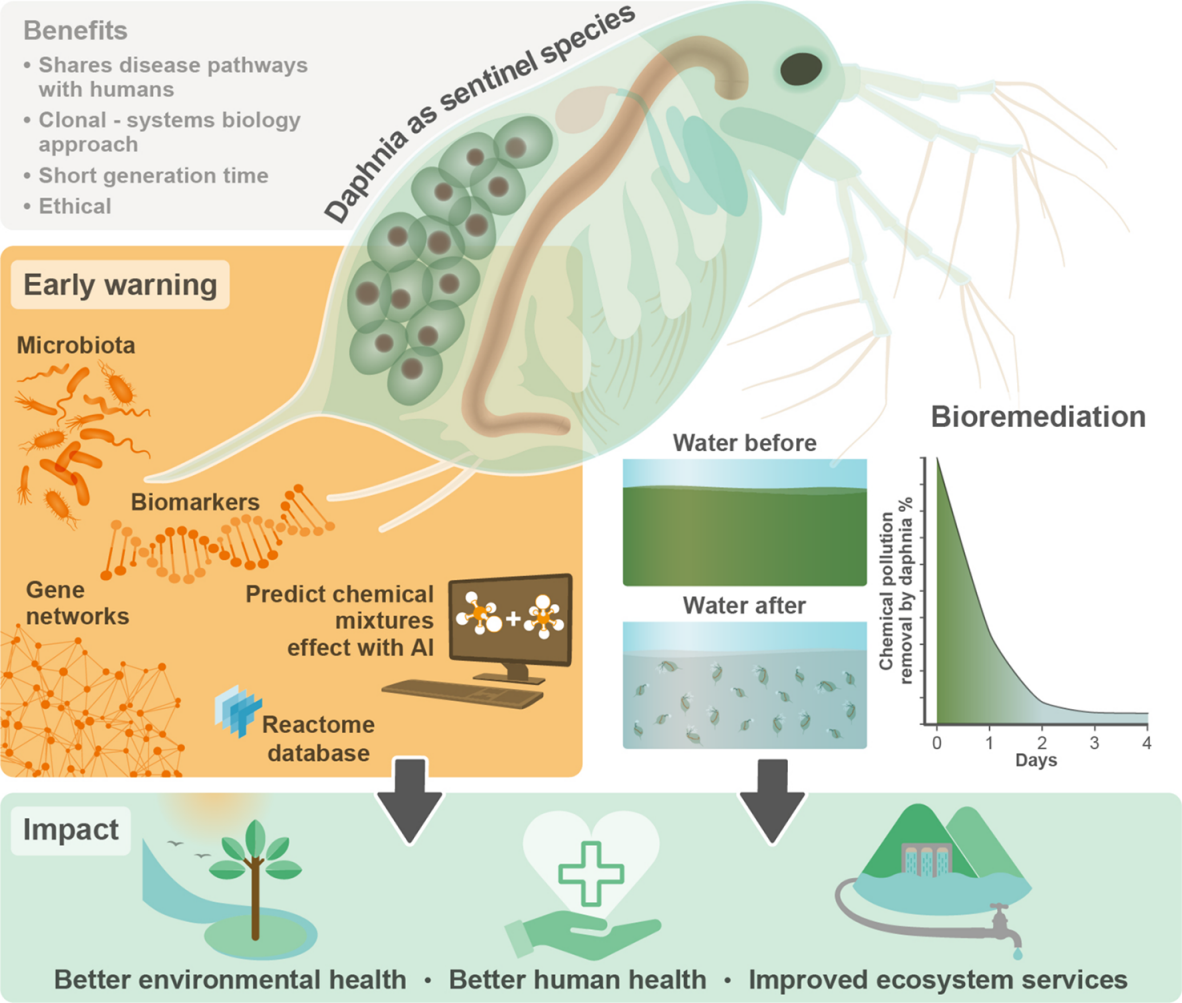
*Daphnia* as an early warning and remedial system. In the proposed framework, the sentinel species *Daphnia* is used both as an early warning system and as a bioremediation tool for chemical pollution. *Daphnia* clonality enables the synchronous analysis of ecological and molecular perturbations by environmental pollution (early warning). This enables the establishment of associations between sublethal doses of chemicals within mixtures and molecular biomarkers. Using the manually curated Reactome database, gene ontologies and conserved molecular functions can be identified in responsive modules across organisms, including humans. Once chemicals have entered the environment, remedial actions are needed. The sentinel species *Daphnia* has the potential to become a sustainable bioremediation agent as it removes excess nutrients from water, preventing eutrophication, and a wide range of persistent chemicals (bioremediation). By using *Daphnia* as a diagnostic and remedial agent, adverse effects for humans and the environment can be significantly reduced (impact).

Model organisms that are distantly related to humans, such as *Drosophila melanogaster* (an insect) and *Caenorhabditis elegans* (a nematode), have historically been used both as surrogates and exemplary models in biomedical research to study fundamental biological processes as well as to understand threats to human health.^18−21^ They are often preferred to mammalian surrogate species for their amenability to experimentation and their 3Rs (replace, reduce, refine) compliance. In addition, they share human disease genes that are ancestral in animal genomes and shared across phylogenetically distant species.^22−24^ The water flea *Daphnia* shares many advantages with these model species. *Daphnia* has a short generation time enabling experimental manipulation of large populations,^25^ has growing genomics resources,^26−29^ and shares many ancestral gene families with humans.^28^*Daphnia* has additional properties that surpass traditional biomedical model species. They (i) have a parthenogenetic life cycle that allows the rearing of genetically identical individuals (clones) from the same genotype, enabling the concurrent quantification of ecological end points and molecular biomarkers using a systems biology approach (e.g., ref (30)), (ii) are keystone species in freshwater food webs and sentinel species for water quality, making them ecological indicators,^31,32^ (iii) are used in regulatory frameworks to set limits on hazardous substances for the environment and are increasingly contributing to new approach methodologies (NAMs) for chemical risk assessments,^33^ and (iv) can biotransform or bioaccumulate chemicals,^34,35^ enabling water bioremediation applications^36^ (Figure 1).

### *Daphnia* as an Early Warning System for Environmental and Human Health

We propose *Daphnia* as a diagnostic early warning system for sublethal effects of chemical pollution in water. This is achieved by measuring exposure-induced biomolecular changes and linking co-response networks of genes and metabolites (hereafter called modules)^37,38^ to the ambient chemical mixtures. These measurements provide a cost-effective way to generate recognizable signatures of chemical exposure that potentially reflect targets of toxicity. Linking molecular-level information to the health of a subject, such as a patient or a surrogate species, is the foundation for precision medicine.^39,40^ “Omics” data are unbiased, providing a global perspective of the molecular biological responses to environmental perturbations without *a priori* knowledge of the potential targets of toxicity.^41^ They also provide an early signature of dose-dependent environmental perturbations, allowing a more nuanced characterization of chemical mixture effects on biological systems.^42,43^ Modules identified with “omics” technologies can be interrogated for their conserved functionality across species based on knowledge of the evolutionary history of genes inherited from a shared common ancestor (i.e., gene orthologs),^44,45^ enabling the prediction of chemical hazard from one species to another (e.g., teratogenicity via aryl hydrocarbon receptor mediated (AhR) pathway activation^46^) and breaking the compartmentalization between human toxicology and ecotoxicology. Furthermore, molecular biomarkers can be useful for the regulatory testing of chemicals, as they have been shown to be predictive of ecological end points, which are typically used for risk assessment.^47^

**Figure 2:**
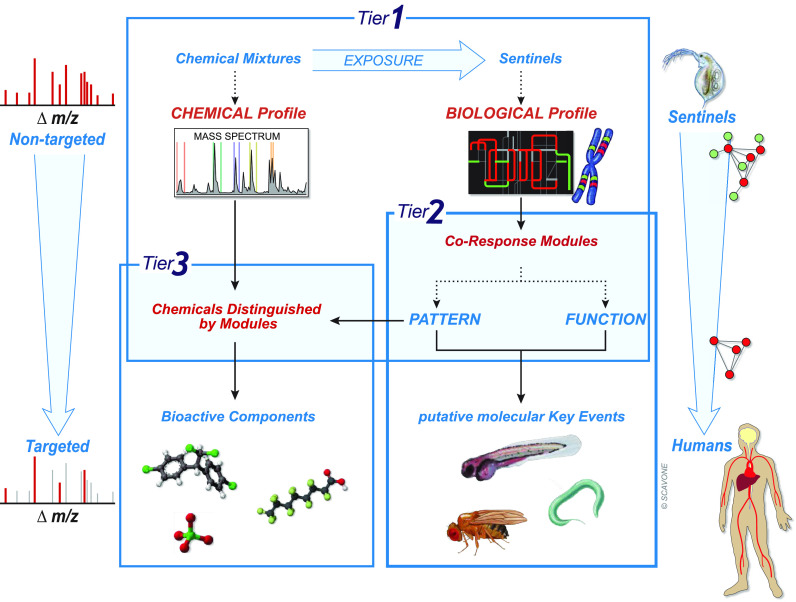
Three-tiered framework. The tiered approach identifies hazards of real-world chemical mixtures with putative molecular key events (putative mKEs) of the sentinel species *Daphnia*. Using functional conservation of gene and metabolite networks, the framework enables the identification of targets of toxicity across species, guiding *in vivo* and *in vitro* validations of toxic effects in human models. The approach consists of three tiers: tier 1 is the nontargeted fingerprinting of real-world environmental mixtures and of the biological effects induced by these mixtures; tier 2 identifies putative mKEs responsive to chemical mixtures; tier 3 establishes associations between bioactive chemical components within the environmental mixtures characterized in tier 1 and putative mKEs identified in tier 2.

We propose a framework that uses nontargeted analysis to characterize real-world chemical mixtures and high-throughput “omics” technologies (e.g., transcriptomics and metabolomics) to identify co-response modules activated by these mixtures. The framework uses orthologs within conserved pathways to enable cross-species extrapolation for the early diagnosis of the potential hazards of chemical pollution, even when chemicals are present at sublethal concentrations. In this framework, *Daphnia* plays the same role that canaries played in coal mines, fulfilling its role as a sentinel species. The approach can also be used for premarket screening of new chemicals and chemical mixtures to improve chemical safety (Figure 2). The framework uses a three-tiered approach, described in the following sections.

#### Tier 1

The sentinel species *Daphnia* is exposed to ambient chemical mixtures (Figure 2, tier 1). Ideally, a nontargeted high-resolution mass spectrometry analysis is used to characterize chemical mixtures that occur in environmental media using the mass-to-charge ratios of ions,^48^ producing a spectrum (chemical profile) of the overall detectable water chemistry. This nontargeted approach enables us to ask not if a specific substance of interest is present in a sample of water, but rather, what is in the water? The nontargeted mass spectrometry analysis can be followed by a targeted analysis to quantify chemical compounds within mixtures.^49−51^ A targeted analysis of multiple compounds (e.g., pharmaceuticals) can be used instead of a nontargeted analysis when the source of contamination is known (see the case study below). The biological effects of the characterized chemical mixtures on the sentinel species *Daphnia* are measured following the OECD 202 guidelines (Figure 2; Figure S1) with the addition of an unbiased screening of the biomolecular responses using “omics” technologies (e.g., transcriptomics and metabolomics as biomolecular profiles).^47,52^

#### Tier 2

The biomolecular response induced by real-world chemical mixtures is measured through transcriptional and/or metabolic coordination among genes/metabolites (features) in co-response modules (Figure 2, tier 2).^53^ The level of the coordinated response of these features is determined with a correlation analysis through Pearson correlation, Spearman’s rank correlation, or mutual information.^54^ The co-response genes and metabolites form coexpression networks, in which highly correlated features form co-response modules that we here call putative molecular key events (putative mKEs)^55,56^ (Figure S1). These putative mKEs are different from the molecular key events (mKEs) in an adverse outcome pathway (AOP) framework,^57^ which are directly linked to an adverse outcome phenotype. Instead, the putative mKEs are biological signatures of chemical exposure that identify putative targets of chemical hazard to be validated experimentally. The genes within the co-response modules are annotated using gene ontologies (e.g., GO^58^). Their functional conservation across species is established using gene orthologies (e.g., orthoDB^59^). The main advantage of using co-response modules based on transcriptional/metabolic coordination is to link unknown to known, as the functions of unannotated genes and metabolites of the co-response modules are inferred based on their membership and coordination within recognizable canonical pathways using the KEGG Network database.^29,53^ The co-response modules are mapped onto biomolecular pathways using KEGG,^60^ PANTHER,^61^ or the Reactome^62^ databases, to name a few, which all use evolutionary and functional classification of genes from organisms across the tree of life. This analysis places orthologs onto pathways and enables the identification of pathways that are evolutionarily conserved among distantly related species. Whereas conservation of pathways does not necessarily mean conservation of mechanisms of toxicity, such conservation provides testable hypotheses to assess conservation of targets of toxicity across species through experimental validation. With functional annotation and pathway information in place, statistical inferences like gene set enrichment analysis (GSEA)^63^ and pathway overrepresentation analysis (POA)^64^ can be performed on each module (Figure S1). The GSEA and POA analyses identify biomolecular pathways that are enriched by genes and metabolites within each module in response to chemical exposure, more than would be expected by chance, providing mechanistic insights into the pathways that are potential targets of toxicity.

#### Tier 3

Significant correlations are identified between the chemical components within real-world mixtures characterized in tier 1 and the co-response modules identified in tier 2 (Figure 2, tier 3). These correlations can be established by matrix-on-matrix regression, also known as multiblock correlation analysis between the omics data (e.g., transcriptomics and metabolomics^65^) and chemical data.^66^ The advantage of this approach is that multiple blocks can be analyzed simultaneously in a single model so that the covarying omics and chemical features among multiple blocks can be identified by machine learning approaches, such as sparse partial least squares discriminate analysis (sPLS-DA^67^), multi-omics factor analysis (MOFA+^68^), and biorder canonical correlation analysis (Bi-CCA^69^). Statistical approaches are then applied to extract significant correlations among the ones identified. The matrix-on-matrix regression is the preferred method for nontargeted data. An alternative approach is the weighted gene coexpression network analysis (WGCNA) that identifies chemical-associated modules using correlation analysis between the eigengene (i.e., the first principal component of a co-response module) and the chemical data.^70^ The eigengene is used as a weighted average value of the gene expression or metabolite profiles in each module.^70^ This approach was used in the case study shown below to validate the framework. When a co-response module that is conserved across species in tier 2 has been associated with a specific chemical, two alternative approaches can be used to assess the hazards on biological systems: (i) the chemical has been previously associated with an adverse phenotype and recorded in the Comparative Toxicology database,^71^ a manually curated database of associations among biomolecular responses and chemicals as identified by experimental evidence; (ii) the correlations identified are novel; therefore, experimental validation is needed to move from correlations to causations. However, the correlation exercise has the main advantage of focusing experimental validations on the putative target of toxicity and on the species in which the targets are conserved. For example, vertebrate surrogate models or human cell lines derived from different tissues may be used to identify adverse effects that are associated with the putative mKEs initially identified in *Daphnia*.

The framework addresses three main outstanding challenges in modern toxicology.

#### Challenge 1: Adversity End Points

Regulations are typically applied to single chemicals and are normally set based on their observed adverse effects from toxicity testing on animals using concentrations that organisms rarely experience in the natural environment.^72,73^ The focus on adversity end points and high chemical doses is logistically advantageous but disregards the effects that may arise from exposures to sublethal doses.^74^ It also fails to identify early warning signatures, which use could presage and thus lead to anticipatory prevention of toxicity end points.^47^ The AOP framework has been a positive step toward the evaluation of toxicity based on the identification of KEs that are predictive of adversity end points. These KEs may be observed as molecular, cellular, structural, or functional changes in biological systems induced by a molecular initiating event, allowing the identification of biomarkers of toxicity.^57^ The important concept introduced by the AOP framework is the clear link between KEs and adverse outcomes at multiple biological levels of organization (e.g., cell, organ, whole organism, ecosystem), including those that are relevant to risk assessment.^57^ Yet, to date, AOPs are not routinely used for risk assessment because they are qualitative.

Our framework identifies putative mKEs activated by exposure to real-world chemical mixtures, often occurring in the environment at sublethal doses, providing targets that are then used to assess exposure hazards to real-world chemical mixtures. As targets of exposure hazard may be indicative of foreseeable toxicity, early biomolecular signatures identified with the approach proposed here can subsequently be linked to biomarkers that are predictive of ecological end points, as previously demonstrated.^47^ Putative mKEs of hazards that are proven to be predictive of adverse phenotypes therefore align with the concept of mKEs in the AOP framework.^75^

#### Challenge 2: Cumulative Effect of Chemical Mixtures

Organisms (including humans) are exposed to intentional and unintentional chemical mixtures; their individual components can be 100-fold below their regulatory approved thresholds and still contribute to the overall toxicity of the mixture (bioactivity).^5,76^ Understanding the cumulative health risks caused by the interaction among chemicals is critical to managing public health and environmental protection. Yet, chemical risk assessment is substance-driven, sector-specific (e.g., pharmaceuticals, cosmetics, and biocides), and prospective—a prospective risk assessment determines, assesses, and minimizes risks before they happen.^5,77^ Whereas intentional mixtures (formulated products) are addressed through a prospective risk assessment prior to the marketing of products, the assessment of accidental chemical mixtures is often limited to combinations of only a small number of compounds. Moreover, chemical safety legislation does not consider exposure to multiple chemicals across sectors (e.g., pharmaceuticals combined with pesticides).^78^

Our framework enables an unbiased hazard assessment of chemical mixtures in water, which consists of chemical pollutants from multiple sources (e.g., domestic, agricultural, industrial), and links the putative mKE to bioactive components within these mixtures, guiding experimental validation of the targets of toxicity. This is a key advance over the current practice to identify the potential hazards of chemical pollution and to generate a testable hypothesis to assess the conservation of targets of toxicity across species. Once these substances are identified, they can then be prioritized for further testing in the context of an adverse outcome pathway and risk management.

#### Challenge 3: Human Toxicology and Ecotoxicology Are Compartmentalized

Risk assessment of toxic substances to humans and the environment has been historically disconnected and compartmentalized. Vertebrate models have been used as surrogates for humans, whereas ecologically relevant algal, invertebrate, and fish species have been used as surrogates for biodiversity.^4^ The use of nonoverlapping surrogate species for human and environmental toxicity has meant that cross-species extrapolation has been applied within human toxicology and within ecotoxicology but not between these compartments.^79^ Cross-species extrapolations made under the testable hypothesis that similarities among species are determined by their shared evolutionary history (i.e., “toxicity by descent”) are being pursued by a European Commission research and innovation funded research program (PrecisionTox; http://precisiontox.org), building on discoveries made in comparative genomics on the ancestry of disease-causing genes in humans.^22^ PrecisionTox employs a suite of biomedical model species for its investigations, including *Daphnia*, and is tasked with addressing the needs for a cohesive approach toward experimental design of a mutually agreed framework to quantitatively identify functionally conserved putative mKEs among species and their links to chemical toxicity.

Our framework enables the identification of bioactive chemicals within environmental mixtures that have a measurable biomolecular effect on the sentinel species *Daphnia*, which may be indicative of hazards to other animals by identifying putative mKEs that are evolutionarily conserved. Our inclusion of an evolutionary analysis of the putative mKEs is a key element for the modern use of *Daphnia* as a sentinel for the protection of other animal species—made possible by a high degree of pathway conservation between invertebrates and humans; human gene sets that already serve as biomarkers of chemical exposure (e.g., the U.S. National Toxicology Program’s s1500+ reference gene panel) are enriched by evolutionarily conserved genes across the animal phylogeny.^80^ The interpretation of these results for extrapolating hazards to other species reasonably assumes that the induction or malfunction of processes that are shared with humans is reflective of the chemical mixtures’ mechanisms of action, but not necessarily predictive of shared adversity. The differences among species in their physiology, organ systems, adaptive responses to exposures, and life history would contribute to differences in such outcomes. However, the application of this new knowledge obtained from our framework can improve environmental health by having clearly defined protection goals. If, for example, the evolutionary analysis of the putative mKEs indicated mutagenesis (or other potential mechanisms of action of greatest concern to chemical hazard assessors), this result would prioritize further investigations potentially leading to regulatory actions. Alternatively, the precautionary principle applies when setting exposure limits in Europe to substances that may cause harm to the environment. The evolutionary analysis of responsive pathways in sentinels that are associated with fundamental biological processes such as reproduction helps guide the application of this principle for protection goals that include biodiversity, while greater scientific knowledge of the actual hazards is obtained. In this way, the evolutionary conservation of response pathways enables the framework to bridge the divide between human toxicology and ecotoxicology.

**Figure 3:**
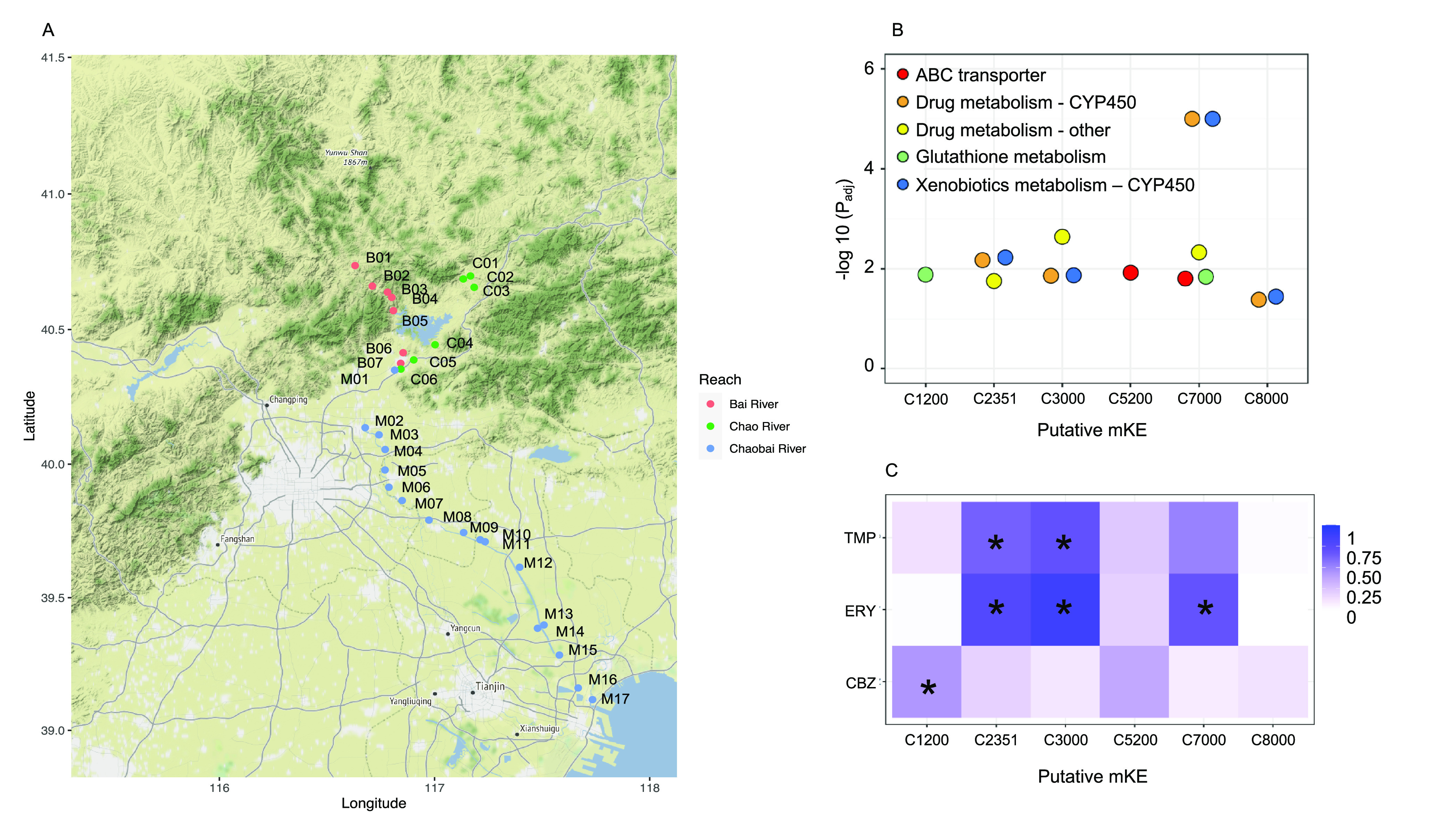
*Daphnia* as a diagnostic early warning system. (A) Map of 30 water sampling sites along the Chaobai river basin, including three main streams (B, Bai river; C, Chao River; M, Chaobai river). These 30 water samples are subjected to targeted chemical analysis to identify 22 organic compounds (mostly pharmaceuticals) listed in Table S1. (B) Six putative mKEs identified via coexpression network analysis, are significantly enriched in xenobiotic and drug metabolic pathways identified in *D. magna* exposed to the Chaobai river water samples. The minus log-transformed adjusted P values are plotted in this figure; (C) Heatmap shows correlations between six putative mKEs and three pharmaceuticals: carbamazepine (CBZ), erythromycin (ERY), and trimethoprim (TMP). The color coding of the correlation coefficient increases from white (0) to dark purple (1), with asterisks (*) marking the significant correlations.

We demonstrate the three-tiered approach in a case study, in which we expose one *Daphnia* strain (IRCHA clone 5; Water Research Centre, Medmenham, U.K.) to water samples collected from 30 sites of the Chaobai river in China (Figure 3A). The river receives industrial and domestic effluent as well as agricultural runoff.^81^ Both the Bai river and the Chao river originate from the Yunwu mountains northeast of Beijing (sites B01–B06 and sites C01–C05; Figure 3). The Chaobai river flows through the urban area north of Beijing and the agricultural area of Tianjin (sites M01–M17; Figure 3). Chemical profiles from the water samples were previously generated using a targeted analysis to identify polar organic pollutants, primarily pharmaceuticals^82^ (Table S1). *Daphnia* were exposed to the Chaobai river waters following the OECD guideline 202 (OECD 202): 24 h old *Daphnia* juveniles were exposed to the river samples without feed for 48 h, and immobilization was recorded using OECD traditional assays. The exposed *Daphnia* were collected after 48 h for total RNA extraction and mRNA sequencing. Methods describing exposures, RNA data generation, and data preprocessing are in the Supporting Information.

The analytical approach described in the framework above was followed for the case study. Immobilization was not observed in any of the exposures. This may have been expected given the sublethal doses of chemical mixtures in the Chaobai river waters. Genes that passed the quality filtering were clustered into co-response modules (putative mKEs) using WGCNA.^70^ Conservation of the genes in these modules between *Dr. melanogaster* and *Daphnia magna* was established using gene orthology within OrthoDB.^83^ The orthologous genes were mapped onto biomolecular pathways using the KEGG PATHWAY database.^60^ From this analysis, a total of 27 co-response modules were identified. A pathway overrepresentation analysis revealed that 6 of the 27 modules were significantly enriched for xenobiotic and drug metabolism functions, namely, xenobiotic/drug metabolism with cytochrome P450 (CYP450), glutathione metabolism, and ABC transporter (Figure 3B). Using a Pearson’s correlation analysis, four modules were identified to be significantly correlated with three pharmaceuticals (i.e., carbamazepine, erythromycin, and trimethoprim, *P*_adj_-value <0.05, corresponding to CBZ, ERY, and TMP in Figure 3C), which were previously detected in the sampled waters.^81^ The enrichment of xenobiotic and drug metabolic pathways that are significantly correlated with pharmaceuticals suggests that the three pharmaceuticals are bioactive and biotransformed by *Daphnia*. However, to establish mechanisms of toxicity for these three compounds within the mixture requires experimental validation, as discussed in the framework above.

Using the KEGG database, we determined that five pathways activated in *Daphnia* by carbamazepine, erythromycin, and trimethoprim were conserved across seven model species (*D. magna*, *Daphnia pulex*, *Danio rerio*, *Dr. melanogaster*, *C. elegans*, *Mus musculus*, and *Homo sapiens*; Table S2). The ortholog compositions of these five pathways were highly conserved in the seven species; *D. magna* shares more than 79% KO (KEGG orthology) terms with other species in four of the five pathways; the ABC transporter pathway is the only one showing lower conservation of orthologs across species, with 54% similarity between *D. magna* and *C. elegans* and 62% similarity between *D. magna* and *Dr. melanogaster* (Table S2). The degree of functional conservation across species suggests that the targets of toxicity are shared across species. However, exposure experiments are needed to determine whether the risk of exposure is also shared.

This case study demonstrates that the three-tiered approach can link bioactive chemicals within mixtures with perturbations of functional pathways, even when adversity end points are not observed, and reveals whether these functional pathways are conserved across species, generating testable hypotheses to identify the targets of toxicity across species. By using *Daphnia* as a “canary in a coal mine”, we can identify putative mKEs activated by real-world chemical mixtures before adverse outcomes occur. Using functional conservation of pathways across species, we can focus experimental validation on potential targets of toxicity in other species, greatly reducing unnecessary experimentation.

### *Daphnia* as a Biobased Solution for Water Bioremediation

Domestic and industrial processes as well as agricultural runoff are the main sources of chemical pollution of surface and wastewater.^84−86^ Once known and unknown chemicals have entered the environment, they are challenging to remove because they are not fully biotransformed or eliminated by current effluent treatments.^87^ Therefore, they end up in downstream waterways where they permeate sediment and soil and bioaccumulate through the trophic chain, eventually causing untoward health effects in humans (e.g., refs (7), (13), and (88)).

Over the last decades, both chemical and mechanical processes have been developed to remove persistent chemicals from effluent water originating from industrial processes, agricultural practices, and human and animal waste, e.g., ref (89). However, these processes have high operational and energy costs, require large infrastructure, and can generate toxic byproducts (e.g., bromate from ozonation for wastewater treatment).^90,91^ Biobased solutions, including phycoremediation, fungal bioremediation, and constructed wetlands (plant bioremediation), are a preferred alternative to current chemical and mechanical processes to meet the net-zero carbon emission and sustainable goals of the international agenda, realized through the European Green Deal, the Zero Pollution Action Plan, and the Chemical Strategy for Sustainability,^77^ and are promising to remediate the environmental impact of pollutants.^92^ However, the removal efficiency of chemicals by emerging biobased solutions is too low for industrial-scale operations, requiring days rather than the needed hours for industrial processes. In addition, biobased solutions can have considerable space and infrastructure requirements (e.g., phycoremediation), demanding significant investment by the private sector and resulting in environmental impact, e.g., ref (92).

We present here for the first time a proof-of-concept study that elevates *Daphnia* to the role of a potential alternative remedial agent for chemical pollution in water and wastewater. First, we benchmark *Daphnia* against other biological agents, i.e., algae and bacteria. Second, we use the properties of *Daphnia* as a fast-evolving organism to environmental pollution^26,27^ to identify strains with higher decontamination abilities that can be tailored to different wastewater sources.

#### Benchmarking

**Figure 4:**
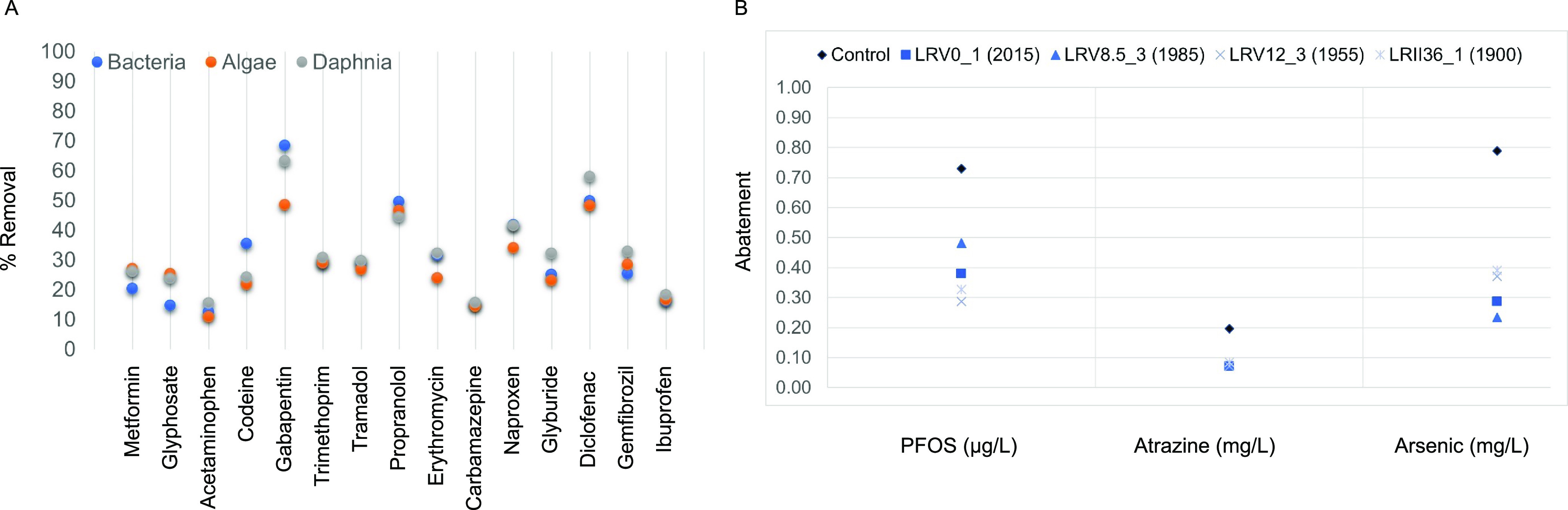
Bioremediation with *Daphnia*. (A) Removal efficiency of known concentrations of 16 pharmaceuticals (ng/L) by bacteria (blue), algae (orange), and *Daphnia* (gray). (B) Abatement of an industrial chemical (PFOS; μg/L), a biocide (atrazine; mg/L), and a heavy metal (arsenic; mg/L) by four strains of *Daphnia* resurrected from a sedimentary archive with different historical environmental backgrounds: LRV0_1 (2015), LRV8.5_3 (1985), LRV12_3 (1955), and LRII36_1 (1900) (ref (26)). The abatement of chemicals is shown after 48 h of exposure and compared to a control—spiked medium without *Daphnia* (control).

Influent tertiary wastewater was collected from the Finham treatment plant in Coventry (U.K.). After collection, the wastewater was equally split in triplicate 20 L aquaria: (i) a first set of aquaria only harbored the naturally occurring bacteria population in the wastewater, (ii) a second set was inoculated with a population of *Daphnia* strains from the stock collection at the University of Birmingham, and (iii) a third set was inoculated with a population of algae (*Chlorella vulgaris*) from the commercially available strain SAG 211/11B. *Ch. vulgaris* is a commonly used bioremediation agent,^93^ shown to remove biocides^94^ and pharmaceuticals,^95^ and is, therefore, a suitable benchmark for *Daphnia*. Following 48 h of exposure, the abatement of 16 pharmaceuticals was quantified as compared to the initial concentrations in the wastewater quantified within 24 h of collection (Table S3). Removal efficiency was calculated as [influent – effluent/influent] × 100. The chemical analysis of target pharmaceuticals was conducted on the biological replicates of influent (reference) wastewater and the experimental aquaria (Table S3). The quantification of the 16 pharmaceuticals was completed using ultraperformance liquid chromatography (UPLC) coupled to a Q-Exactive Orbitrap high-resolution mass spectrometer following ref (96). Following solid-phase extraction (SPE) of target pharmaceuticals from wastewater samples, both acidic and basic pharmaceuticals were determined using rapid polarity switching electrospray ionization sources. Full scan MS mode at a resolution of 35 000 fwhm and an automatic gain control (AGC) target of 1 × 10^6^ ions at an injection time of 50 ms provided the optimum parameters for high sensitivity, together with sufficient data points per peak (≥15) for improved reproducibility. A high-resolution accurate mass with a low mass tolerance filter (<5 ppm from authentic standards) was applied to achieve maximum selectivity with method limits of detection ranging between 0.02 and 1.21 ppb. *Daphnia* removed 7 of the 16 pharmaceuticals more efficiently than algae and bacteria and the remaining 9 pharmaceuticals at a comparable rate (Figure 4A; Table S3).

#### Strain-Specific Chemical Removal

Having assessed that *Daphnia* survives in wastewater and abates chemicals better or equally well than other biological agents, we then tested the removal efficiency of different *Daphnia* strains in a first effort to identify strains with higher decontamination abilities. This is relevant because different wastewater sources may contain different chemical cocktails. We capitalize on our previous work in which we studied fitness responses of four *Daphnia* strains resurrected from a sedimentary archive of a lake with a well-known history of chemical pollution.^26,97^ The lake experienced no chemical exposure until the 1970s and high chemical exposure from 1975 onward. In our previous work, we showed that genotypes that were historically exposed to chemical stress showed reduced genomewide diversity and lower fitness when exposed to novel chemical stress. This lower fitness was underpinned by reduced gene diversity at detoxification, catabolism, and endocrine genes. These results suggest potential lower tolerance to novel chemical stress and higher tolerance to recurring stress in experienced genotypes.^26^ Here, we study the removal efficiency of these strains—two that were naïve to chemical stress (LRV12_3 and LRII36_1) and two that experienced historical chemical stress (LRV0_1 and LRV8.5_3; Figure 4)—following 48 h of exposure to a metal (arsenic), a biocide (atrazine), and an industrial chemical (PFOS).^26^ On the basis of historical records, all strains were naïve to arsenic, whereas LRV_1 and LRV8.5_3 were likely pre-exposed to atrazine and PFOS, even if empirical estimates of these compounds in the lake are not available.

All chemical measurements of water exposed to the four *Daphnia* strains were done in technical duplicates. Atrazine was analyzed according to the method described above by Abdallah et al.,^96^ while PFOS was quantified using the method reported by Harrad et al.^98^ Briefly, water samples were extracted by SPE using Oasis-WAX cartridges (6 mL, 150 mg, Waters). PFOS was quantified on a Sciex Exion UPLC coupled to a Sciex 5600+ TripleTOF mass spectrometer (MS). The TripleTOF MS is equipped with a Turbo V ion source operated in negative mode using electrospray ionization at a voltage of −4500 V and operated at 450 °C. Mass spectrometric data was acquired using automatic information-dependent acquisition (IDA) with a dependent product ion scan using a collision energy of −40 V. The method detection limit for PFOS was 0.5 ppb. Arsenic samples were prepared using 50 ppb germanium as the internal standard. Samples prepared with 70% nitric acid were incubated at 20 °C for 18 h, vortex-mixed for 30 s, and 100 μL aliquots were diluted to 10 mL using DI water. The samples were quantified using a Nexion 300X inductively coupled plasma mass spectrometer (ICP–MS) (PerkinElmer, Seer Green, U.K.) fitted with a cyclonic spray chamber. Calibration curves spanning 1–20 ppb were constructed in DI water.

On average, *Daphnia* removed 47.3% of PFOS, 60% of atrazine, and 60% of arsenic. However, the strains had different removal efficiencies across the three chemicals, with a maximum removal of 59% for PFOS (LRV12_3), 65% for atrazine (LRV_1 and LRV8.5_3), and 70.7% for arsenic (LRV8.5_3) (Figure 4B; Table S4). The removal efficiency observed in this study, considering the fitness response to chemical exposure observed in our previous study, suggests that a strain’s removal efficiency is likely influenced by historical exposure to chemicals. Strains historically exposed to chemical stress (e.g., LRV_1 and LRV8.5_3) show a higher removal efficiency to atrazine and arsenic. Conversely, LRV12_3 that is naïve to PFOS showed the highest removal efficiency. These results support our previous conclusions that strains may evolve tolerance to recurring but not novel stress. However, we previously showed that higher tolerance is associated with lower genomewide diversity. If the patterns observed in the strains used here are validated at the population level, they suggest that acquired tolerance to chemical stress is evolutionarily advantageous to recurring but not novel chemical stress and comes at a cost.^26^

This proof-of-concept study shows that *Daphnia* has the potential to become a systemic solution for the removal of a wide range of persistent chemicals from water, preventing their diffusion through other environmental matrixes (e.g., soil) and their bioaccumulation through the trophic chain. The ability to tailor strains of *Daphnia* to different wastewaters is a powerful way to tackle different contamination sources. With additional optimization, the *Daphnia*-based removal of chemicals from wastewater can meet the requirement of the water industry for residence times of a few hours. Additionally, whereas photobioreactors using algae need a large infrastructure due to their residence time,^95^*Daphnia* populations can be retrofitted within tertiary treatment tanks. To contain the *Daphnia* populations, containment devices can be used that allow the flow-through of water while containing the *Daphnia* population.^36^ Prototype filtration devices (details are not disclosed because they are commercially sensitive) have been designed to retain stable *Daphnia* populations, prevent live animals from escaping from the containment volume, and collect dead *Daphnia* postfiltration.^36^ The dead animals are siphoned into a biowaste treatment process where extant technologies (e.g., oxidative catalysis) proven for other biowastes can destroy residual contaminants accumulated in the *Daphnia* body, preventing bioaccumulation and biomagnification.^99^ While substantial work is required to translate this proof-of-concept bioremediation solution into a market-ready technology, the use of the sentinel species *Daphnia* to remediate the effect of chemicals in the aquatic environment has the potential to maximize the shift to clean growth, enabling water reuse, reducing resource depletion and environmental pollution, and sustaining vital ecosystem services.

## Conclusions and Future Research Needs

The proposed framework has the potential to improve both the detection and the mitigation of environmental chemical pollution with a single sentinel species. The use of sentinel species to identify evolutionarily conserved pathways perturbed by the same chemicals across the tree of life is potentially transformative to identify targets of toxicity while reducing unnecessary vertebrate animal testing. Whereas experimental validation is required to establish causation between chemicals/chemical mixtures and adversity end points, the framework guides experimental efforts capitalizing on the evolutionary conservation of pathways. The use of advanced computational approaches, such as machine learning, to identify correlations between chemicals within mixtures and putative mKEs can significantly improve environmental protection by identifying functional pathways that may lead to adversity before major harm happens. The ability to identify chemicals within mixtures that have an adverse outcome enables greater precision in the regulation of chemicals as mixtures based on real-world environmental exposure.

*Daphnia* can also work as a sustainable bioremediation solution once chemical mixtures have entered the environment and remediation is the only solution. Some level of pollution is likely unpreventable, so effective bioremediation tools will always be needed. By expanding the use of *Daphnia* both as an early warning diagnostic and a remedial tool, we address challenges associated with the entire life cycle of chemicals and their mixtures.

National and international regulatory bodies will be understandably cautious in adopting the proposed framework. The framework must be shown to be cost-effective, to provide enhanced protection of human and environmental health, to be usable by regulators and industries, and to be comprehensible by the public. This will take time and a period of transition where it is tested, validated, and accepted. Major EU initiatives, such as the Zero Pollution Action Plan, linked three ongoing EU projects on new approach methodologies (NAMs) to form a cluster called ASPIS, whose main goal is to improve chemical safety without animal testing (https://chemicalwatch.com/370080/eu-non-animal-projects-brought-together-for-aspis-cluster). These initiatives clearly indicate a desire for novel approaches for assessing and managing chemical pollution. The transition to the novel methodologies proposed here will require changes in regulatory frameworks, following a test and acceptance phase. This will take time and resources, but the potential benefits for human and environmental health will justify the effort.

The framework can be, in principle, extended to other model species with the advantage of improving our understanding of targets of toxicity in multiple species. The use of multiple animal models and of human cell lines within the same framework can help distinguish evolutionarily conserved biomarkers across the tree of life from biomarkers that only affect certain taxonomic groups, focusing regulatory interventions where they are most needed with reduced impact on industrial production and other human activities.

## Notes

Transcriptomics data supporting the Chaobai river case study are openly available in NCBI at accession no. PRJNA809147.

## Supporting Information Available

Step-by-step analytical pipeline of the proposed framework (PDF)
Supporting methods for the Chaobai river case study, organic pollutants in the Chaobai river, KEGG pathways identified in the Chaobai river study and conserved across species, removal of 16 pharmaceuticals by different biological agents, abatement of three chemicals by different *Daphnia* strains, and step-by-step analytical pipeline of the proposed framework (PDF)
