# Supplementary figures and images for: Daphnia as a Sentinel Species for Environmental Health Protection: A Perspective on Biomonitoring and Bioremediation of Chemical Pollution
Source: Environ Sci Technol. 2022 Sep 28;56(20):14237–48. doi: 10.1021/acs.est.2c01799 (PMC9583619; doi:10.1021/acs.est.2c01799)

**Tier 1: Nontargeted profiling**

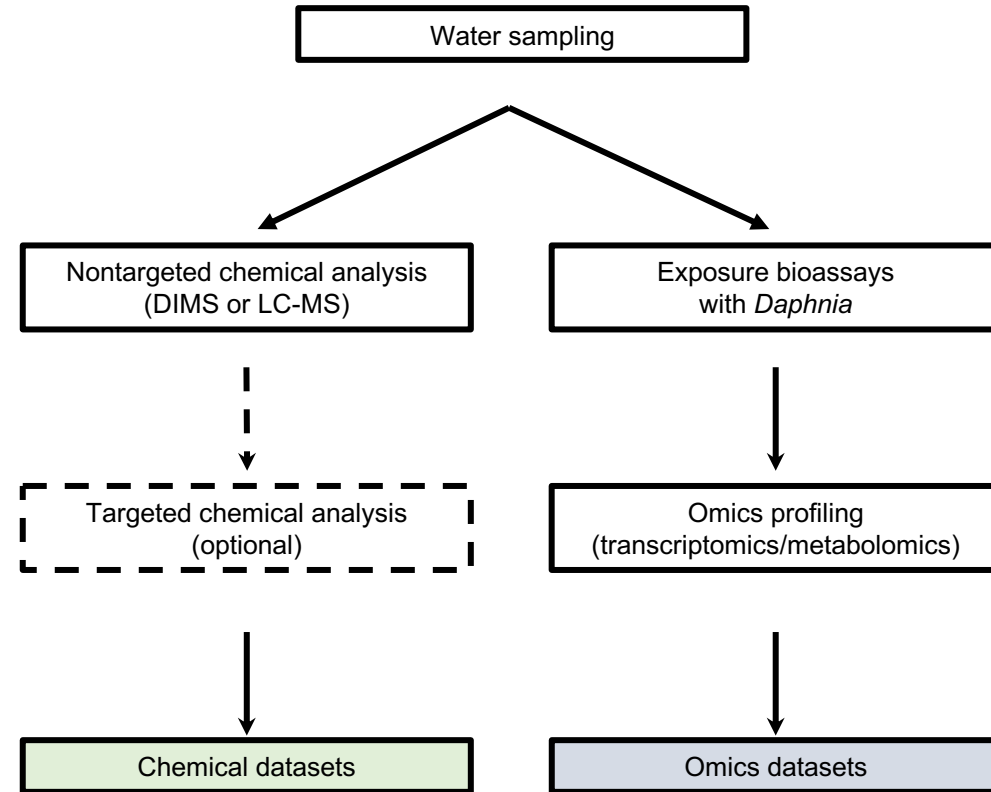

**Tier 2: Co-response modules**

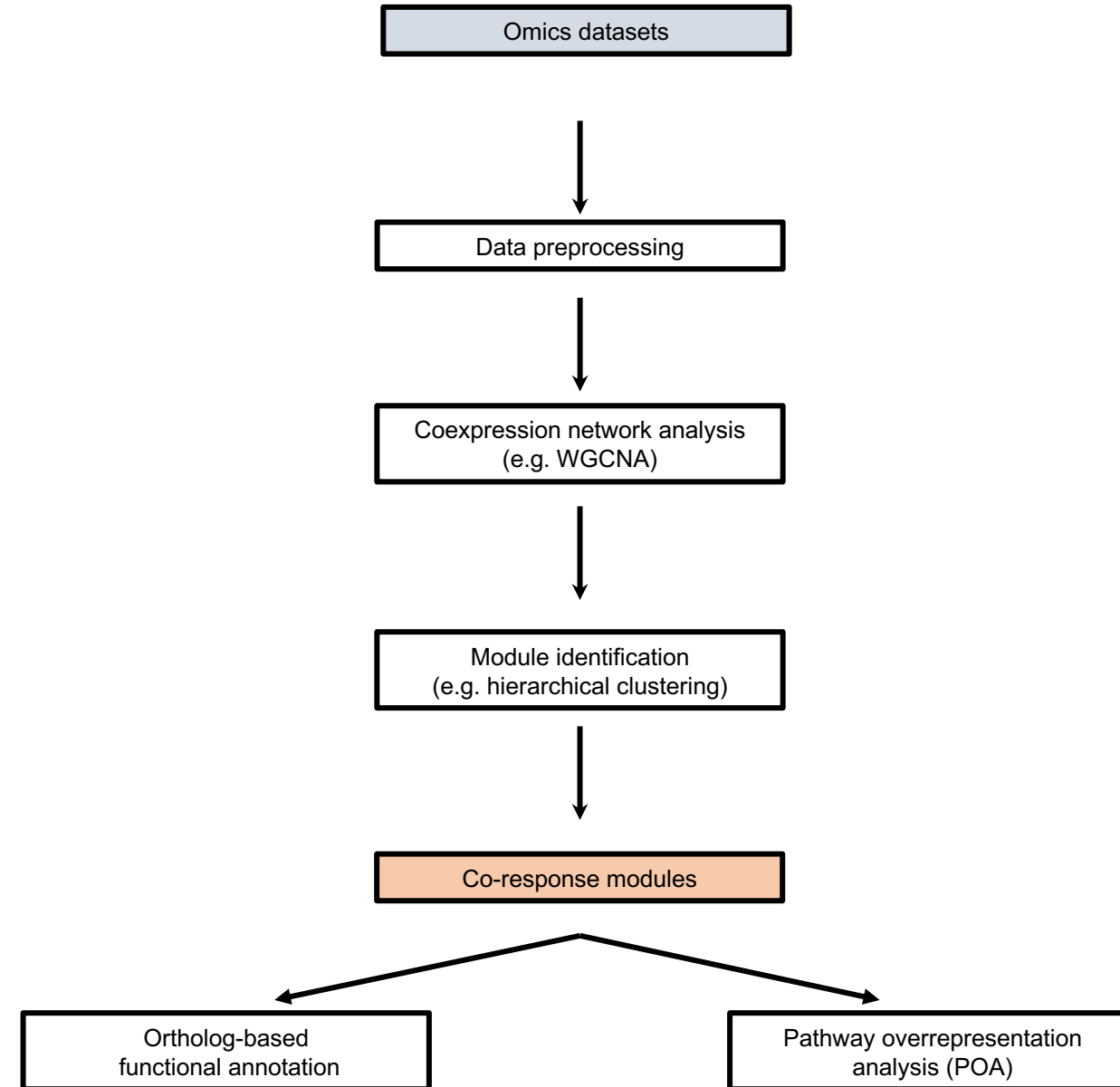

**Tier 3: Chemical-module correlations**

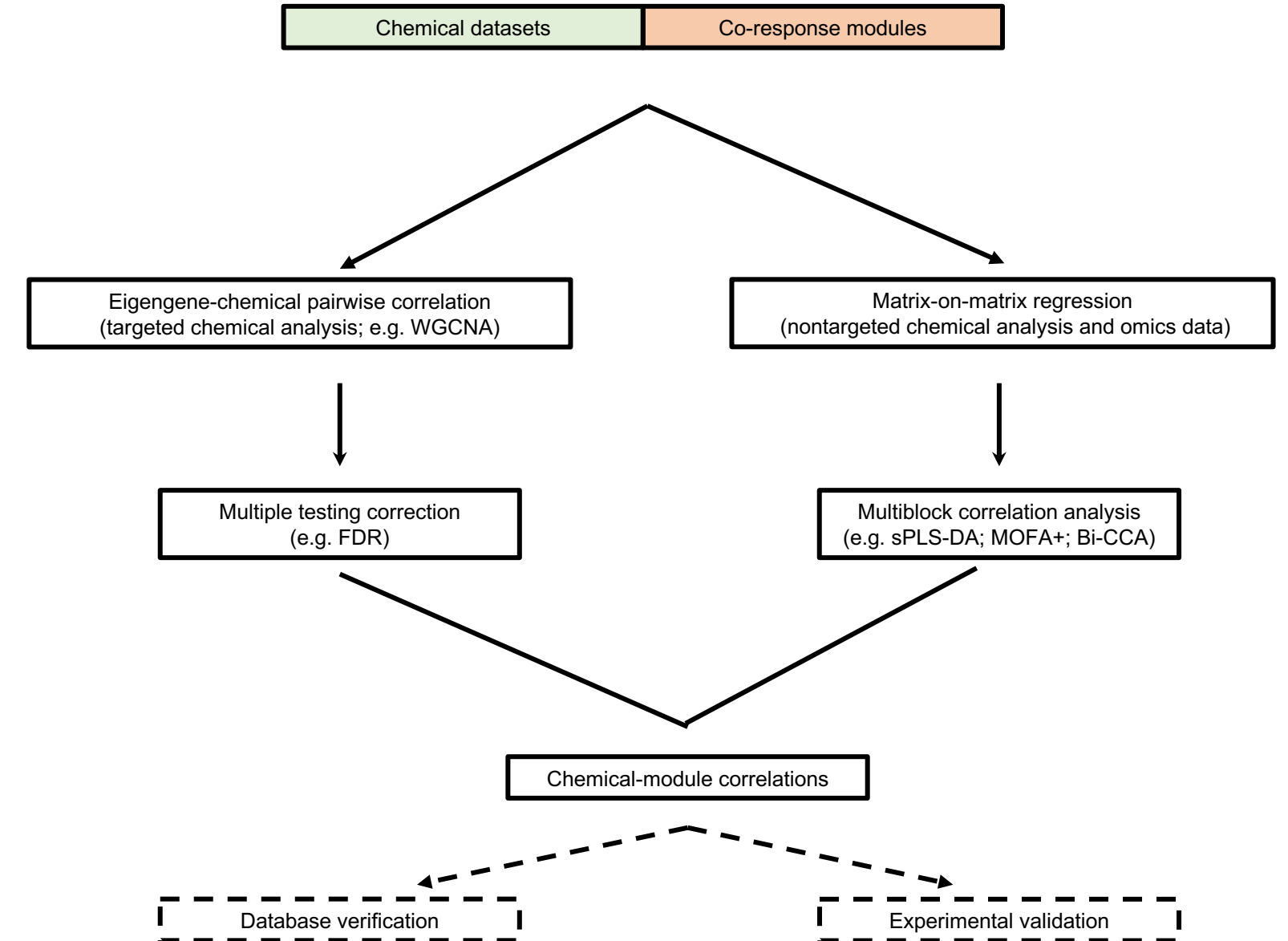

Supplement: Supplementary file 1 — es2c01799_si_001.pdf [file es2c01799_si_001.pdf]
