# Supplementary material for: Daphnia as a Sentinel Species for Environmental Health Protection: A Perspective on Biomonitoring and Bioremediation of Chemical Pollution
Source: Environ Sci Technol. 2022 Sep 28;56(20):14237–48. doi: 10.1021/acs.est.2c01799 (PMC9583619; doi:10.1021/acs.est.2c01799)

Daphnia as sentinel species for environmental health protection: a perspective on biomonitoring and bioremediation of chemical pollution

Muhammad Abdullahi[1*], Xiaojing Li[1*], Mohamed Abou-Elwafa Abdallah[2], William Stubbings[2], Norman Yan[3], Marianne Barnard[1], Liang-Hong Guo[4], John K. Colbourne[1§] and Luisa Orsini[1,5§]

[1]Environmental Genomics Group, School of Biosciences, the University of Birmingham, Birmingham B15 2TT, U.K.

[2]School of Geography, Earth and Environmental Sciences, the University of Birmingham, Birmingham B15 2TT, U.K.

[3]Department of Biology, York University, and Friends of the Muskoka Watershed, Bracebridge, Ontario P1L 1T7, Canada

[4] Institute of Environmental and Health Sciences, China Jiliang University, 258 Xueyuan Street, Hangzhou, Zhejiang 310018, People's Republic of China

[5]The Alan Turing Institute, British Library, 96 Euston Road, London NW1 2DB, U.K.

*Shared first authorship

[§] Shared senior authorship

Supporting information: 7 pages

SI methods: Supporting methods for the Chaobai river case study

SI Table 1: Organic pollutants in the Chaobai river

SI Table 2: KEGG pathways identified in the Chaobai river study and conserved across species

SI Table 3: Removal of 16 pharmaceuticals by different biological agents

SI Table 4: Abatement of three chemicals by different *Daphnia* strains

SI Figure 1: Step-by-step analytical pipeline of the proposed framework

Supporting methods for the Chaobai river case study

Daphnia magna 24h-old juveniles (IRCHA clone 5; Water Research Centre, Medmenham, UK) were exposed to 30 water samples from the Chaobai river in triplicates. The exposure assays followed the OECD 202 guidelines. After 48 h of exposure, immobilization was recorded, and mobile juveniles were flash frozen for total RNA extraction and mRNA sequencing from exposed *Daphnia* and from clonal replicates maintained in control conditions. Total RNA was extracted using the RNA Advance Tissue kit (Beckman Coulter) applied to flash-frozen tissue following the manufacturer's instructions. Extracted RNA was quantified using a Nanodrop-8000 Spectrophotometer (ThermoFisher ND-8000-GL) and integrity assessed on the Agilent Tapestation 2200 (Agilent G2964AA) with High Sensitivity RNA Screen Tapes (Agilent 5067-5579). Total RNA (1µg) was poly(A) selected using the NEBNext® Poly(A) mRNA Magnetic Isolation Module (New England Biolabs E7490L) and then converted in mRNA libraries using a NEBNext Ultra Directional RNA Library Prep Kit (New England Biolab E7420L) and NEBnext Multiplex Oligos for Illumina Dual Index Primers (New England Biolabs E7600S), following the manufacturer guidelines. Sample handling was performed with the Biomek FxP workstation (Beckman Coulter A31842). Constructed libraries were assessed for quality using the Tapestation 2200 (Agilent G2964AA) with High Sensitivity D1000 DNA Screen Tape (Agilent 5067-5584). Multiplexed libraries (100-bp paired end) were sequenced on a HiSeq4000 by the Beijing Genomics Institute (BGI) to obtain 5M reads per sample. Sequenced reads quality was assessed using fastqc (v0.11.5) [1], followed by multiqc (v1.5) [2]. Transcripts were mapped onto the *D. magna* reference transcriptome [3, 4] using default settings in Salmon (version 0.8.2). The reads were then trimmed using Trimmomatic 0.32 [5] with the following parameters: (i) Illumina adapter cutoff with two seed mismatches, (ii) palindrome clip threshold of 30 and a simple clip threshold of 10, (iii) Phred quality score >30, (iv) minimum trimmed reads length of 50 bp. The read count matrix of mapped transcripts was summarised at gene level and further analysed in R (version 4.0.3). Low count genes (genes with read count < 10/sample) were removed. Read counts were normalised by the size factor defined in the DESeq2 package (version 1.30.0; [6]). A total of 14,705 genes were clustered on co-responsive modules using WGCNA [7] to identify 27 co-responsive modules or putative molecular key events (mKEs). For each putative mKE, we identified orthologous groups between *Drosophila melanogaster* and *D. magna* using OrthoDB [8]. Ortholog were mapped onto functional pathways using the KEGG pathway database [9]. Pathway overrepresentation analysis was done using the Fisher's exact test. Correlations between chemical components withing mixtures and eigengenes (the first principal component) of co-response modules are depicted using the Pearson correlation coefficients where the P-value are adjusted by a Benjamini-Hochberg procedure (P_{adj}-value < 0.05). The general workflow of data analysis is illustrated in Figure S1. Pathway conservation between *Daphnia magna* and six other model species (*Daphnia pulex*, *Danio rerio*, *Drosophila melanogaster*, *Caenorhabditis elegans*, *Mus musculus*, and *Homo sapiens*) is assessed using the KEGG orthology (KO) requested from the KEGG PATHWAY database. The composition of KOs of the five pathways mentioned in the case study (i.e., ABC transporter, drug metabolism – cytochrome P450, drug metabolism – other, glutathione metabolism, xenobiotic metabolism – cytochrome P450) in *Daphnia magna* are compared with the composition of KOs in six other species, where the number and percentage of shared KOs are recorded in Table S2.

References

1. Brown, J.; Pirrung, M.; McCue, L. A., FQC Dashboard: integrates FastQC results into a web-based, interactive, and extensible FASTQ quality control tool. *Bioinformatics* **2017,** *33*, (19), 3137-3139.
2. Ewels, P.; Magnusson, M.; Lundin, S.; Kaller, M., MultiQC: summarize analysis results for multiple tools and samples in a single report. *Bioinformatics* **2016,** *32*, (19), 3047-8.
3. Orsini, L.; Brown, J. B.; Shams Solari, O.; Li, D.; He, S.; Podicheti, R.; Stoiber, M. H.; Spanier, K. I.; Gilbert, D.; Jansen, M.; Rusch, D. B.; Pfrender, M. E.; Colbourne, J. K.; Frilander, M. J.; Kvist, J.; Decaestecker, E.; De Schamphelaere, K. A. C.; De Meester, L., Early transcriptional response pathways in Daphnia magna are coordinated in networks of crustacean-specific genes. *Mol Ecol* **2018,** *27*, 886–897.
4. Orsini, L.; Gilbert, D.; Podicheti, R.; Jansen, M.; Brown, J. B.; Solari, O. S.; Spanier, K. I.; Colbourne, J. K.; Rusch, D. B.; Decaestecker, E.; Asselman, J.; De Schamphelaere, K. A.; Ebert, D.; Haag, C. R.; Kvist, J.; Laforsch, C.; Petrusek, A.; Beckerman, A. P.; Little, T. J.; Chaturvedi, A.; Pfrender, M. E.; De Meester, L.; Frilander, M. J., Daphnia magna transcriptome by RNA-Seq across 12 environmental stressors. *Sci Data* **2016,** *3*, 160030.
5. Bolger, A. M.; Lohse, M.; Usadel, B., Trimmomatic: a flexible trimmer for Illumina sequence data. *Bioinformatics* **2014,** *30*, (15), 2114-20.
6. Love, M. I.; Huber, W.; Anders, S., Moderated estimation of fold change and dispersion for RNA-seq data with DESeq2. *Genome Biol* **2014,** *15*, (12), 550.
7. Langfelder, P.; Horvath, S., WGCNA: an R package for weighted correlation network analysis. *BMC Bioinformatics* **2008,** *9*, 559.
8. Zdobnov, E. M.; Kuznetsov, D.; Tegenfeldt, F.; Manni, M.; Berkeley, M.; Kriventseva, E. V., OrthoDB in 2020: evolutionary and functional annotations of orthologs. *Nucleic Acids Res* **2021,** *49*, (D1), D389-D393.
9. Kanehisa, M.; Araki, M.; Goto, S.; Hattori, M.; Hirakawa, M.; Itoh, M.; Katayama, T.; Kawashima, S.; Okuda, S.; Tokimatsu, T.; Yamanishi, Y., KEGG for linking genomes to life and the environment. *Nucleic Acids Res* **2008,** *36*, (Database issue), D480-4.
10. Su, D.; Ben, W.; Strobel, B. W.; Qiang, Z., Occurrence, source estimation and risk assessment of pharmaceuticals in the Chaobai River characterized by adjacent land use. *Sci Total Environ* **2020,** *712*, 134525.
11. Mi, H.; Muruganujan, A.; Ebert, D.; Huang, X.; Thomas, P. D., PANTHER version 14: more genomes, a new PANTHER GO-slim and improvements in enrichment analysis tools. *Nucleic Acids Res* **2019,** *47*, (D1), D419-D426.
12. Jassal, B.; Matthews, L.; Viteri, G.; Gong, C.; Lorente, P.; Fabregat, A.; Sidiropoulos, K.; Cook, J.; Gillespie, M.; Haw, R.; Loney, F.; May, B.; Milacic, M.; Rothfels, K.; Sevilla, C.; Shamovsky, V.; Shorser, S.; Varusai, T.; Weiser, J.; Wu, G.; Stein, L.; Hermjakob, H.; D'Eustachio, P., The reactome pathway knowledgebase. *Nucleic Acids Res* **2020,** *48*, (D1), D498-D503.

Supplementary Table 1. List of organic pollutants and their concentration range in water samples from the Chaobai River as reported in ref 10. The site names are corresponding to those in Figure 3A. The compound names and abbreviations; the CAS numbers (CAS No.); the limit of quantification of each compound (LOQ; ng/L); the concentration range reported by Su *et al.* (ref 122) for each compound in the Chaobai River basin, with the site with the highest concentration in parentheses; the number of sites at which the chemical was detected (above LOQ) are shown in this table.

**Compound (abbreviation)**	**CAS No.**	**LOQ (ng/L)**	**Range (ng/L) (site)**	**No sites**
Atenolol (ATE)	29122-68-7	1.82	0-5.84 (M06)	1
Azithromycin (AZN)	83905-01-5	0.34	0-4.99 (M06)	3
Bezafibrate (BF)	41859-67-0	0.41	0-10.86 (M06)	13
Caffeine (CAF)	58-08-2	1.40	0-64.69 (M06)	29
Carbamazepine (CBZ)	298-46-4	0.36	0-35.23 (M11)	25
Clarithromycin (CLA)	81103-11-9	0.74	0-4.60 (M06)	3
Erythromycin (ERY)	114-07-8	0.86	0-593.68 (M16)	27
Metoprolol (MET)	37350-58-6	1.08	0-52.73 (M06)	10
Roxithromycin (ROX)	80214-83-1	0.63	0-29.48 (M06)	5
Sulfadiazine (SDZ)	68-35-9	1.31	0-22.62 (M06)	4
Sulfamethoxazole (SMX)	57-68-1	1.37	0-260.20 (M06)	12
Trimethoprim (TMP)	738-70-5	0.69	0-132.18 (M16)	16
Ciprofloxacin (CIP)	85721-33-1	0.79	0-7.42 (C03)	3

Chlortetracycline (CTC)	64-72-2	0.99	0-1.58 (M08)	1
Doxycycline (DOX)	564-25-0	1.03	0-18.69 (M17)	5
Enrofloxacin (ENR)	93106-60-6	0.55	0-6.62 (C03)	6
Lomefloxacin (LOM)	98079-51-7	0.52	0-5.09 (C03)	2
Norfloxacin (NOR)	70458-96-7	1.12	0-3.87 (C06)	1
Oxytetracycline (OTC)	79-57-2	0.93	0-2.09 (B07)	1
Propranolol (PROP)	526-66-6	0.66	0-4.99 (M12)	1
Sulfamerazine (SMR)	127-79-7	1.62	0-4.96 (C06)	1
Tetracycline (TET)	60-54-8	1.37	/	0

Supplementary Table 2. KEGG pathways conserved across species based on the KEGG orthology (KO) between *Daphnia magna* and six model species (*Daphnia pulex*, *Danio rerio*, *Drosophila melanogaster*, *Caenorhabditis elegans*, *Mus musculus*, and *Homo sapiens*). The total number of orthologous groups (and the percentage over the total ortholog group within a given pathway) shared between *D. magna* and other species are shown.

**Pathway ID**	**map00480**	**map00980**	**map00982**	**map00983**	**map02010**
Pathway Description	Glutathione metabolism	Metabolism of xenobiotics by cytochrome P450	Drug metabolism - cytochrome P450	Drug metabolism - other enzymes	ABC transporters
No orthologs in *Daphnia magna*	21	5	6	19	13
No orthologs shared with *Daphnia pulex*	19 (90%)	5 (100%)	6 (100%)	19 (100%)	12 (92%)
No orthologs shared with *Danio rerio*	19 (90%)	4 (80%)	5 (83%)	19 (100%)	12 (92%)
No orthologs shared with *Drosophila melanogaster*	18 (86%)	4 (80%)	5 (83%)	15 (79%)	8 (62%)
No orthologs shared with *Caenorhabditis elegans*	19 (90%)	5 (100%)	5 (83%)	17 (89%)	7 (54%)
No orthologs shared with *Mus musculus*	20 (95%)	5 (100%)	6 (100%)	19 (100%)	13 (100%)
No orthologs shared with *Homo sapiens*	20 (95%)	5 (100%)	6 (100%)	19 (100%)	13 (100%)

Supplementary Table S3. Concentration (ng/L) of 16 pharmaceuticals in wastewater at the time of sampling (Reference) and following treatment with Bacteria, Algae or *Daphnia*. For each treatment, three biological replicates were generated (R1, R2, and R3) with ultraperformance liquid chromatography (UPLC), coupled to a Q-Exactive™ Orbitrap high resolution mass spectrometer. This table supports Figure 4A in the main manuscript file. Abbreviation: Conc., concentration; AVE, average.

**Conc. (ng/L)**	**Reference**				**Bacteria**				**Algae**				***Daphnia***			
**Chemical name**	**R1**	**R2**	**R3**	**AVE**	**R1**	**R2**	**R3**	**AVE**	**R1**	**R2**	**R3**	**AVE**	**R1**	**R2**	**R3**	**AVE**
Metformin	658	784	742	728	563	549	628	580	517	582	494	531	506	568	539	538
Glyphosate	82	107	76	88	78	72	76	75	63	69	66	66	63	75	64	67
Acetaminophen	890	768	819	826	709	715	743	722	708	763	737	736	667	681	744	697
Codeine	197	234	153	195	128	119	130	126	149	138	170	152	140	172	131	148
Gabapentin	36	32	27	32	14	7	9	10	11	15	23	16	9	12	14	12
Trimethoprim	417	387	462	422	323	301	277	300	291	310	297	299	301	285	291	292
Tramadol	436	388	476	433	303	326	288	306	311	338	302	317	309	283	322	305
Propranolol	36	21	42	33	18	17	15	17	18	15	20	18	21	16	18	18
Erythromycin	76	58	59	64	47	49	36	44	56	45	46	49	50	39	42	44
Carbamazepine	806	761	784	784	686	641	664	664	682	656	674	671	672	680	633	662
Naproxen	128	147	154	143	90	76	84	83	88	93	102	94	79	90	82	84
Glyburide	303	268	243	271	199	216	195	203	228	216	182	209	197	174	181	184
Ibuprofen	10783	11023	10881	10896	9172	8869	9370	9137	9514	8633	9156	9101	9017	8952	8773	8914
Diclofenac sodium	121	129	92	114	58	54	60	57	59	56	62	59	52	53	39	48
Gemfibrozil	743	858	785	795	539	581	661	594	574	612	522	569	521	553	530	535

Supplementary Table S4. Controlled laboratory exposures of four *Daphnia magna* strains (LRV0_1; LRV8.5_3; LRV12_3; and LRII36_1) to PFOS (µg/L), atrazine (mg/L) and arsenic (mg/L). Influent is the concentration of each compound (note the different units) spiked in the growth medium and effluent is the final concentration of each chemical after exposure to *D.magna* for 48 h. Control refers to spiked medium without *D.magna*. This table supports Figure 4B in the main manuscript file.

PFOS (µg/L)	Influent	Effluent
Control	0.73	0.67
LRV0_1	0.73	0.38
LRV8.5_3	0.73	0.48
LRV12_3	0.73	0.29
LRII36_1	0.73	0.33
Atrazine (mg/L)		
Control	0.18	0.20
LRV0_1	0.18	0.07
LRV8.5_3	0.18	0.08
LRV12_3	0.18	0.07
LRII36_1	0.18	0.08
Arsenic (mg/L)		
Control	0.79	0.79
LRV0_1	0.79	0.29
LRV8.5_3	0.79	0.23
LRV12_3	0.79	0.37
LRII36_1	0.79	0.39

Figure S1. Step-by-step analytical pipeline for the proposed framework. In tier 1 water samples are collected from different sources. A nontargeted chemical analysis is applied to the water samples to quantify chemical mixtures, optionally followed by a targeted chemical analysis. *Daphnia* are exposed to the water samples in a battery of OECD bioassays, at the end of which tissues are collected for omics data analysis. Biochemical matrices are the output of tier 1. In tier 2, coexpression network analysis (e.g. WGCNA [7]) is applied to omics data to identify co-response modules. The KEGG [9], Panther [11] and Reactome database [12] are then used for functional annotation of these modules. Enrichment of response modules within functional pathways is achieved with a pathway overrepresentation analysis (POA). In tier 3, correlations between co-response modules identified in tier 2 and chemicals in mixtures identified in tier 1 are established. These correlations can be established following two analytical processes: (i) matrix-on-matrix regression analysis with machine learning to establish significant correlations, which is preferred for nontargeted data; and (ii) correlation between the first principal component of co-expression module (eigengene) and targeted chemical analysis data using in WGCNA pipeline. Once significant correlations are established between modules and chemicals, these can be validated through search in public databases (if they are already known) or experimentally (if they are novel). This figure complements Figure 2 in the main text.

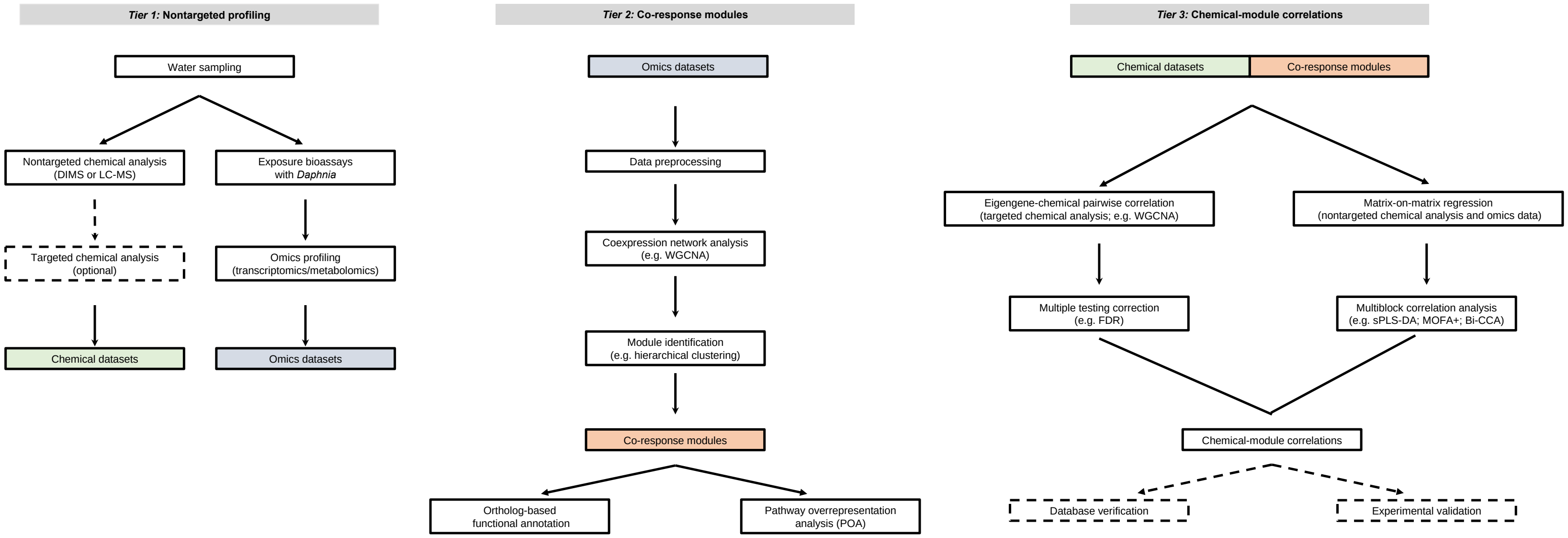

Supplement: Supplementary file 2 — es2c01799_si_002.pdf [file es2c01799_si_002.pdf]
